# Priority setting for the implementation of effective interventions for cancer prevention through collaboration among patients, the public and researchers in Japan

**DOI:** 10.1186/s12885-026-16415-2

**Published:** 2026-06-30

**Authors:** Mai Yamaguchi, Mariko Nishihara, Yuki Kaji, Megumi Yamashita, Miyuki Odawara, Junko Saito, Asako Mimura, Chikako Yamaki, Manami Inoue, Tomio Nakayama, Taichi Shimazu

**Affiliations:** 1 Division of Behavioral Sciences, National Cancer Center Institute for Cancer Control, National Cancer Center, Tokyo, Japan; 2 Graduate School of Public Health, St Luke’s International University, Tokyo, Japan; 3 Graduate School of Public Health, Teikyo University, Tokyo, Japan; 4 Division of Cancer Information Service, National Cancer Center Institute for Cancer Control, National Cancer Center, Tokyo, Japan; 5 Division of Prevention, National Cancer Center Institute for Cancer Control, National Cancer Center, Tokyo, Japan; 6 Division of Screening Assessment and Management, National Cancer Center Institute for Cancer Control, National Cancer Center, Tokyo, Japan

**Keywords:** Cancer prevention, Implementation science, Research priorities, Priority setting

## Abstract

**Background:**

Despite the availability of effective interventions for cancer prevention, Japan faces substantial evidence–practice gaps, such as in the low utilization of smoking cessation treatments, HPV vaccination, and cancer screening. Implementation research plays a critical role in bridging this gap by promoting the uptake of effective interventions in real-world settings. Globally, however, prioritization of the implementation of these various interventions remains unclear. To address this need, we conducted a structured priority-setting exercise using the CHNRI method and incorporating perspectives from diverse stakeholders, including citizens.

**Methods:**

We conducted a four-phase research prioritization process using the CHNRI method to identify which effective interventions for cancer prevention should be prioritized for implementation in Japan. The process included listing effective interventions for cancer prevention, surveying stakeholders, conducting a web-based survey among researchers, and calculating priority scores.

**Results:**

Among 215 invited researchers, 92 (42.8%) and 67 (31.2%) researchers responded to the first and second surveys, respectively. In both surveys, physicians accounted for the largest proportion of respondents. Across both surveys, HPV vaccination for females, cervical cancer screening, fecal occult blood testing (FOBT), mammography, and tobacco control policies were identified as top priorities.

**Conclusions:**

Priorities for implementing effective interventions for cancer prevention were set through collaboration with researchers and citizens, providing a foundation for future research and progress.

**Supplementary Information:**

The online version contains supplementary material available at https://doi.org/10.1186/s12885-026-16415-2.

## Background

In Japan, it has been estimated that 36% of cancer incidences and 41% of cancer-related deaths are attributable to preventable risk factors [1]. Cancer is the leading cause of death, with lung, colorectal, gastric, and pancreatic cancers accounting for a substantial proportion of the overall burden. Compared with Western countries, the incidence of lung cancer in men and cervical cancer in women remains relatively high, whereas obesity-related cancers are comparatively less prevalent. This pattern suggests that, relative to Western countries, cancer risk factors in Japan may be driven less by obesity-related dietary and lifestyle factors and more by infection-related factors [2]. Notably, however, many of these cancers are preventable through established interventions, such as tobacco control and vaccination; or can be detected early through screening, including for colorectal and breast cancer [2]. Despite these opportunities for prevention and early detection, however, the uptake of key interventions remains suboptimal in Japan. The utilization of smoking cessation treatments and the uptake of the HPV vaccine remain low in Japan. Similarly, the uptake of colorectal, breast, and cervical cancer screening is lower than in other OECD countries [3]. This situation exists even though smoking cessation treatments are covered by national health insurance and these cancer screening programs are available at low cost through municipal subsidies. These observations indicate a marked evidence–practice gap in cancer prevention in Japan, and ensuring that effective interventions for cancer prevention reach the public is a pressing issue in public health.

Implementation science and research aims to promote the uptake of evidence-based interventions (EBIs) into real-world practice and contributes to decreasing evidence–practice gaps [4]. It offers a structured approach to identifying barriers and facilitators (context of implementation settings) for implementing EBI, implementation strategies (packaged implementation interventions to address barriers in the context of ensuring implementation success), and implementation outcomes (process outcomes of implementation, which indicate implementation success) [5].

Successful implementation of EBIs under real-world resource constraints requires that the available EBIs be prioritized [6]. For example, the U.S. Cancer Moonshot initiative established a Blue Ribbon Panel that identified ten recommendations to accelerate cancer research. Among these, the dissemination and expansion of scientifically proven cancer prevention and early detection interventions, such as HPV vaccination, colorectal cancer screening, and tobacco control policies, were emphasized [7]. However, the Blue Ribbon Panel consisted solely of experts, and its decisions were not derived through a transparent or reproducible process. Moreover, it remains unclear how such a transparent and reproducible prioritization process for cancer prevention EBIs can be established across diverse contexts, such as different countries, regions, and health system settings. Resources are limited not only for the implementation of EBIs but also for the research needed to support the scaling up of implementation. Researchers and policymakers have increasingly recognized the critical importance of developing such a prioritization process. Furthermore, engaging a broad range of stakeholders not limited to researchers is essential to optimizing the relevance, legitimacy, and acceptability of the identified priorities [8].

The Child Health and Nutrition Research Initiative (CHNRI) method is an established approach for setting priorities in health research [9]. It is considered a systematic and transparent method, as it enables the structured listing and scoring of a large number of research questions based on predefined criteria, while also aggregating the perspectives of diverse experts and stakeholders. Due to its high flexibility, the CHNRI method has been used in a wide range of fields, including child health and nutrition, climate change and health, infectious diseases, and non-communicable diseases (NCDs), as well as at multiple levels, from national initiatives to international organizations [10].

In recent years, patient and public involvement (PPI)—in which researchers collaborate with patients and members of the public from the earliest stages of study planning—has gained increasing attention not only as a means of ensuring research ethics and transparency, but also as a key driver for translating research findings into practice. In Japan also, PPI has gradually gained traction. A recent scoping review by Mathieson et al. (2025) reported that, in many studies, involving citizens in the design and implementation of programs enhanced participants’ understanding and acceptance, and thereby improved program sustainability [11]. As the importance of inclusive and structured priority-setting grows, the need for a structured approach to setting priorities for both research and implementation is pressing.

In this context, we conducted a structured four-phase research prioritization process using the CHNRI method to identify which effective cancer prevention interventions should be prioritized for implementation in Japan. In Japan, cancer control is guided by The 4th Basic Plan to Promote Cancer Control Programs, Japan, which outlines overall goals and strategic directions. However, the prioritization of specific interventions and the processes by which such priorities are determined are not always explicitly articulated. Against this background, we considered that our study may contribute to cancer control planning in Japan by identifying priority areas for implementation research in cancer prevention, and help strengthen the prioritization of interventions within existing national strategies. Further, in the context of constrained public health resources and persistent implementation gaps in cancer prevention, we also considered that our study may support the more strategic allocation of implementation efforts, in both Japan and other countries.

## Methods

The research prioritization process was conducted in four phases.

### Phase 1: listing of effective interventions for cancer prevention recommended for implementation in Japan

We developed a list of effective interventions for cancer prevention recommended for implementation in Japan (Table 1). We selected recommendations from the United States Preventive Services Task Force (USPSTF) and Community Preventive Services Task Force (CPSTF) as reference sources because both task forces specialize in preventive interventions, provide clearly defined, cancer prevention–specific recommendations, and offer consistent grading of evidence for each intervention. Frameworks such as the WHO “Best Buys” and Europe’s Beating Cancer Plan were not used as primary sources, as our study required intervention-specific recommendations with systematically graded evidence for cancer prevention implementation.

**Table 1:** List of effective interventions for cancer prevention recommended for implementation in Japan

Category	Intervention	Details/Notes	Reference
Tobacco Use	Tobacco control policies	Legislation, taxation, advertisement restrictions	Cancer Prevention Recommendation for Japanese
	Smoking cessation treatment	Provided through outpatient clinics	
	School-based smoking prevention education	Targeting adolescents	
Alcohol Consumption	Screening for unhealthy alcohol use in adults aged 20 and over, and brief behavioral counseling to reduce it		Cancer Prevention Recommendation for Japanese
	Price increase on alcoholic beverages to reduce alcohol consumption		CPSTF
Physical Activity/Obesity Prevention	Weight-loss interventions for obese adults	BMI ≥ 30; ≥5% reduction over 1–2 years via diet and exercise counseling	USPSTF
	Community-wide physical activity campaigns	Media, education, local health events	CPSTF
	Environmental improvements for physical activity	Access to facilities, infrastructure, land-use planning	CPSTF
Infectious Disease Prevention	HBV/HCV screening	Blood test (general population and during pregnancy)	Cancer Prevention Recommendation for Japanese
	HBV vaccination	Routine immunization	
	HPV vaccination	Routine immunization	
	H. pylori infection screening	Blood, urine, or stool test	
Cancer Screening	Colorectal cancer screening	Fecal occult blood test (FOBT)	Guidelines for Priority Health Education on Cancer Prevention and Implementation of Cancer Screening
	Gastric cancer screening	X-ray or endoscopy	
	Breast cancer screening	Mammography	
	Cervical cancer screening	Pap smear and/or HPV test	
	Lung cancer screening	X-ray	
	Lung cancer screening	Low-dose CT for eligible heavy smokers	
High-risk Populations	Genetic counseling/testing for HBOC	Risk assessment, BRCA1/2 testing	
	Chemoprevention for high-risk breast cancer individuals	Use of preventive medications	USPSTF

*Abbreviations*: *USPSTF* United States Preventive Services Task Force, *CPSTF* Community Preventive Services Task Force, *HBV* Hepatitis B virus, *HCV* Hepatitis C virus, *HPV* Human papillomavirus, *FOBT* Fecal occult blood test, *HBOC* Hereditary breast and ovarian cancer

For primary prevention, we included interventions which targeted risk factors for cancers identified in the Cancer Prevention Recommendation for Japanese issued by the National Cancer Center Institute for Cancer Control, National Cancer Center, Japan [12].

For secondary prevention, we included the five cancer screening programs recommended by the Japanese government regardless of whether they were endorsed by the USPSTF or CPSTF. These were implemented in accordance with screening guidelines developed by the Institute for Cancer Control of the National Cancer Center, Japan [13]. Details of the guideline development process have been described elsewhere [14]. The programs were included in the list due to their established relevance within Guidelines for Priority Health Education on Cancer Prevention and Implementation of Cancer Screening [15].

### Phase 2: stakeholder survey of a patient-public panel

In this study, a Patient-Public Panel organized by the Institute for Cancer Control, National Cancer Center, Japan participated as a key stakeholder. This panel was established in 2008 to incorporate the perspectives of patients and the public into cancer control in Japan. It consists of approximately 100 members recruited through an open call from across the country, each of whom serves a two-year term. Members are selected based on predefined eligibility criteria, including experience or interest in cancer-related issues and the ability to engage with diverse stakeholders. They contribute their insights and perspectives based on their experiences as patients or family members, as well as their interests, occupations, social positions, and the characteristics of their local communities [16].

#### Pre-meeting

Prior to the meeting, a pre-meeting questionnaire was distributed to each member of the Patient-Public Panel. The survey asked about the participant’s personal experiences with interventions for cancer prevention, and which intervention they believed should or should not be implemented, along with the reasons (evaluation criteria) behind their views. Responses were received from 86 of the 100 panel members. Participants were asked to assess the following seven interventions selected from a list developed in Phase 1 of the study, focusing on those deemed relatively familiar to patients and members of the public as it had already been adopted at the policy level (1-6), or its adoption had been decided (7): (1) tobacco control through policy measures; (2) smoking cessation treatment via outpatient clinics; (3) hepatitis B virus (HBV) and hepatitis C virus (HCV) screening (blood tests); (4) human papillomavirus (HPV) vaccination; (5) Helicobacter pylori infection testing; (6) the five cancer screening programs recommended by the government (colorectal cancer [fecal occult blood test], gastric cancer [barium X-ray or endoscopy], breast cancer [mammography], cervical cancer [Pap test and HPV test], and lung cancer [chest X-ray and sputum cytology for heavy smokers]); and (7) Low-dose computed tomography (CT) screening for lung cancer in heavy smokers. Three evaluation criteria were provided as options: “burden of disease reduction,” “equity,” and “cost”, but new criteria such as “equality” and “cost-effectiveness” also emerged from the open-ended responses.

#### Meeting

The panel members meet twice a year in hybrid meetings (both online and in-person) to facilitate dialogue. For the present study, researchers explained to the panel during one of these meetings that, from an implementation science perspective, prioritization is essential for effectively delivering effective interventions for cancer prevention to the public, especially under conditions of limited resources and personnel. They also noted that transparent systems to incorporate citizens’ perspectives into such prioritization processes are needed in Japan. During the meeting discussion, “time to observable impact” (i.e., how quickly an intervention yields observable outcomes) was also proposed as an additional evaluation criterion.

#### Post-meeting

Following the meeting, a post-meeting survey was conducted. Based on the meeting discussion, the descriptions of the evaluation criteria were revised to improve clarity. Participants were then asked to rank nine evaluation criteria in order of perceived importance when determining priorities for effective interventions for cancer prevention at the national, prefectural, or municipal level. Of the 70 meeting participants, 57 responded to the post-meeting questionnaire. Results are shown in Table 2.

**Table 2:** Ranking of evaluation criteria for prioritizing effective interventions for cancer prevention to implement answered by the Patient-Public Panel/ researchers

Criterion	Description	Rank answered by the Patient-Public Panel	Rank answered by researcher
Effectiveness (Reduction in Disease Burden)	Whether the intervention can substantially reduce cancer incidence and mortality at the national or local level when widely adopted.	1	1
Perceived Effectiveness (Observability)	Whether the effectiveness of the intervention is tangible or observable in everyday life. Example: Seeing someone benefit from early cancer detection through screening and successful treatment.	2	7
Reduction in Disparities (Equity)	Whether the intervention helps reduce disparities in cancer incidence and mortality associated with socioeconomic status, education, or geographic location. Example: Subsidizing screening costs for low-income individuals to reduce mortality disparities.	3	8
Simplicity of Use (Low Complexity)	Whether the intervention is simple and convenient for users, with minimal procedures required. Example: Municipalities offering multiple cancer screenings in one coordinated visit.	4	3
Gap Between Evidence and Practice	Whether there is a significant gap between known effective interventions for cancer prevention and their actual use in the population. Example: Low uptake of cancer screening or low follow-up rates after positive screening results.	5	2
Low Financial Burden	Whether the intervention can be implemented at a relatively low cost to national or local governments, enabling widespread access. Example: Providing cancer screening at minimal cost to the municipality.	6	6
Cost-Effectiveness	Whether the benefits of the intervention justify the implementation costs. Example: Colorectal cancer screening that significantly reduces mortality for a reasonable public investment.	7	5
Uniform Access (Equality in Provision)	Whether the intervention is provided under the same conditions for all individuals across the population. Example: Equal subsidies for cancer screening offered to all residents.	8	Not measured
Short Time to Observable Impact	Whether the benefits of the intervention —such as reductions in cancer incidence or mortality—become evident at the population level in a relatively short period after implementation.	9	9
Feasibility	The potential for preventive interventions to be broadly operationalized within public health programs, policies, and clinical practices	Not measured	4

### Phase 3: web survey of researchers

We identified researchers conducting cancer prevention studies from a public health perspective (i.e., at the population level) through a review of databases containing English-language publications and publicly funded research project reports. To obtain a broad perspective, we did not restrict selection to researchers in the specific domains of cancer prevention. For English-language publications, a search was conducted in PubMed to identify observational and interventional studies related to primary and secondary cancer prevention among the Japanese population published over the past five years (from 2020 to November 15, 2024). This search yielded 276 articles. Two independent reviewers (M.N. and Y.K.) screened the titles and abstracts of these articles for suitability, and any discrepancies were resolved through discussion within the research team. As a result, 142 articles were selected for inclusion.

Regarding publicly funded research, cancer prevention-related projects funded between 2020 and 2024 by the Ministry of Education, Culture, Sports, Science and Technology (MEXT), the Ministry of Health, Labour and Welfare (MHLW), and the Japan Agency for Medical Research and Development (AMED) were extracted, resulting in 39 identified projects.

After excluding duplicates and the principal investigator (T.S.) of the present study from the list of corresponding authors of the selected publications and the principal and co-investigators of the publicly funded projects, a total of 215 unique researchers were identified as the target population for this study. Invitations to participate in the survey were sent either to the email addresses provided in published articles or by postal mail to the institutional affiliations publicly available on the researchers’ websites. No personal or otherwise identifiable information was collected through the survey.

#### First survey

The first survey served as a preparatory step for the prioritization process, with two main objectives: (1) to complete the list of effective interventions for cancer prevention made in Phase 1 by incorporating additional items suggested by survey participants; and (2) to assess the perceived importance of evaluation criteria considered for use in prioritizing these interventions in the second survey.

To complete the list, participants were invited to suggest additional items. Based on the responses, three additional items were added to the list of interventions for cancer prevention: (1) cancer education for elementary, junior high, and high school students, as specified in Japan’s national curriculum guidelines; (2) HPV vaccination for males, as recommended by the U.S. Centers for Disease Control and Prevention (CDC); and (3) dissemination of knowledge on primary cancer prevention, guided by Japan’s Cancer Control Act and the Basic Plan to Promote Cancer Control Programs. The results of the first researcher survey are detailed in Additional file 1.

To assess the perceived importance of evaluation criteria, participants were asked to rate the importance of each evaluation criterion in prioritizing interventions for cancer prevention using a five-point scale, with responses of “very important,” “important,” “neither important nor unimportant,” “not very important,” and “not important at all.” The evaluation criteria were further refined based on feedback from a post-meeting survey conducted with the Patient-Public Panel (Phase 2). “Feasibility” was explicitly incorporated, while “equity” was excluded due to feedback indicating that its distinction from “reducing disparities” was unclear to some respondents. As a result, the five evaluation criteria rated as most important were: (1) reduction in disease burden (2), evidence-practice gap (3), simplicity (4), feasibility, and (5) cost-effectiveness. In contrast to the Patient–Public Panel, which ranked observability and equity second and third, researchers assigned these items markedly lower positions, at seventh and eighth, respectively (Table 2). However, since the list of effective interventions for cancer prevention included those not yet implemented in Japan, the criterion “evidence-practice gap” was excluded from the final set. The Patient-Public Panel survey did not include “feasibility” as a separate item because it was considered difficult for the general public to assess, as it refers to the feasibility of implementing a preventive measure broadly across programs, policies, and clinical practice. Therefore, we merged the criteria “simplicity” and “feasibility” into a single criterion labeled “feasibility” in the list of criteria for the second survey. Furthermore, “reduction of disparities,” which was ranked as the third-most important criterion in the Patient-Public Panel survey, was added to the list of criteria for the second survey.

#### Second survey

The second survey was conducted to evaluate candidate cancer prevention interventions using predefined evaluation criteria identified by both researchers and the Patient-Public Panel. This process enabled the calculation of scores for each intervention, which informed their prioritization in subsequent phases. In the second researcher survey, the same 215 researchers from the first survey were asked to evaluate each cancer prevention strategy using the following criteria: (1) the extent to which nationwide implementation in Japan could contribute to a reduction in disease burden (2), its cost-effectiveness, (3) its potential to reduce disparities, and (4) its overall feasibility. For each criterion, respondents were asked to indicate whether the intervention had a high, neutral, or low potential, or to select “Do not know” if they were unsure.

### Phase 4: calculation of priority scores

Following previous studies that applied the CHNRI method to derive weighted priority scores [9], we first calculated unweighted priority scores for each intervention deemed important for implementation. For each evaluation criterion, responses were scored as “High importance” = 1 point, “Neutral” = 0.5 points, and “Low importance” = 0 points. The total score for each intervention was then divided by the number of valid responses (excluding those who selected “Don’t know”) to obtain an unweighted priority score.

Subsequently, we applied a weighting procedure using the results of the post-meeting questionnaire conducted with the Patient-Public Panel. In that survey, participants were asked to rank the importance of four evaluation criteria used in the second researcher survey. The results indicated the following order of relative importance: (1) reduction in disease burden (2), reduction of disparities (3), feasibility, and (4) cost-effectiveness.

In the CHNRI method, when assigning weights to evaluation criteria based on their priority, a weighting coefficient (W) is calculated using the following formula [9]:

$$\textrm{W}=\textrm{Ideal average rank}/\textrm{Observed average rank}$$

The derived weights were then applied to the unweighted scores to calculate the final weighted priority score for each cancer prevention.

## Results

Respondents to the researcher survey are characterized in Table 3. A total of 92 responses were obtained for the first researcher survey (response rate: 43%) and 67 for the second (response rate: 31%). In both surveys, the majority of respondents were male (62.0% in the first survey, 65.6% in the second), and approximately 70% had over 15 years of research experience. The most common professional background was physician (65.2% in the first survey, 59.7% in the second), followed by nurse or public health nurse (9.8% in the first survey, 13.4% in the second). Regarding institutional affiliation, the most frequently represented institutions were medical faculties at universities (45.7% in the first survey, 40.3% in the second), followed by nursing faculties (10.9% and 14.9%, respectively) and research institutes (13.0% and 13.4%, respectively).

**Table 3:** Characteristics of the researchers’ survey respondents

		First survey (*N* = 92)	Second survey (*N* = 67)
Sex	Male	57 (62.0%)	44 (65.6%)
	Female	35 (38.0%)	23 (34.3%)
Institutional affiliation	University (Faculty of Medicine)	42 (45.7%)	27 (40.3%)
	University (Faculty of Nursing)	10 (10.9%)	10 (14.9%)
	University (School of Public Health)	1 (1.2%)	1 (1.5%)
	University (Other)	10 (10.9%)	7 (10.4%)
	Research Institution	12 (13.0%)	9 (13.4%)
	Medical Checkup Center	2 (2.2%)	3 (4.5%)
	Hospital (excluding university hospital)	10 (10.9%)	6 (9.0%)
	National or local government	2 (2.2%)	0 (0.0%)
	Other	3 (3.3%)	4 (6.0%)
Years of research experience	< 5 years	6 (6.5%)	4 (6.0%)
	≥ 5 years and < 15 years	20 (21.7%)	15 (22.4%)
	≥ 15 years	66 (71.7%)	48 (71.6%)
Occupation and Qualifications	Physician	60 (65.2%)	40 (59.7%)
	Nurse/Public Health Nurse	9 (9.8%)	9 (13.4%)
	Pharmacist	3 (3.3%)	2 (3.0%)
	Clinical Laboratory Technologist	3 (3.3%)	3 (4.5%)
	Registered Dietitian/Dietitian	4 (4.4%)	2 (3.0%)
	Physical/Occupational/Speech Therapist	0 (0.0%)	0 (0.0%)
	Not applicable	13 (14.1%)	11(16.4%)

Results of the second researchers’ survey are shown in Table 4. Regardless of whether weighting was applied, the top five prioritized interventions for cancer prevention were: (1) HPV vaccination for females (2), cervical cancer screening (3), FOBT for colorectal cancer (4), mammography for breast cancer screening, and (5) tobacco control policies. Table 5 presents rankings of effective interventions for cancer prevention by each evaluation criterion without weighting. HPV vaccination for females was identified as the intervention most likely to contribute to both a reduction in disease burden and reduction in disparities. Tobacco control policies were perceived as the most cost-effective intervention, while FOBT for colorectal cancer was rated as the most feasible.

**Table 4:** Priority score of effective interventions for cancer prevention recommended for implementation in Japan

Rank	Effective interventions for cancer prevention	Weighted priority score	Unweighted priority score
1	HPV vaccination for females	1.18	0.88
2	Cervical cancer screening	1.15	0.87
3	FOBT for colorectal cancer	1.13	0.87
4	Mammography for breast cancer screening	1.12	0.84
5	Tobacco control policies	1.11	0.84
6	HBV/HCV screening during pregnancy	1.10	0.83
7	Cancer education for elementary, junior high, and high school students (including smoking prevention education)	1.05	0.78
8	HBV vaccination	1.02	0.78
9	H. pylori infection screening (blood, urine, or stool test)	1.02	0.76
10	HBV/HCV screening (blood test)	1.00	0.75
11	HPV vaccination for males	0.96	0.70
12	Raising awareness of knowledge related to primary cancer prevention	0.95	0.71
13	Smoking cessation treatment in clinics and support for quitting smoking in health checkups and medical settings	0.92	0.68
14	Gastric cancer screening (X-ray or endoscopic examination)	0.91	0.68
15	Cancer risk assessment, genetic counseling, and genetic testing for patients and relatives with HBOC	0.87	0.61
16	Low-dose CT lung cancer screening for heavy smokers meeting specific criteria	0.84	0.58
17	Increasing the price of alcoholic beverages to reduce alcohol consumption	0.82	0.61
18	Screening for unhealthy alcohol use in adults aged 20 and over, and brief behavioral counseling to reduce it	0.78	0.55
19	Lung cancer screening (X-ray)	0.78	0.61
20	Community-wide campaigns to increase physical activity	0.72	0.54
21	Environmental improvements to promote physical activity	0.71	0.50
22	Weight loss interventions for obese adults	0.69	0.46
23	Prescription of preventive medication for individuals at high risk of breast cancer, such as those with a family history	0.66	0.46

*Abbreviations*: *HBV* Hepatitis B virus, *HCV* Hepatitis C virus, *HPV* Human papillomavirus, *FOBT* Fecal occult blood test, *HBOC* Hereditary breast and ovarian cancer, *CT* Computed Tomography

**Table 5:** Rankings of effective interventions for cancer prevention by individual evaluation criteria without weighting

Rank	Reduction in disease burden	Cost-effectiveness	Reduction of disparities	Feasibility
1	HPV vaccination for females	Tobacco control policies	HPV vaccination for females	FOBT for colorectal cancer
2	Cervical cancer screening	FOBT for colorectal cancer	Cervical cancer screening	HBV/HCV screening during pregnancy
3	Tobacco control policies	HPV vaccination for females	HBV/HCV screening during pregnancy	Mammography for breast cancer screening
4	Mammography for breast cancer screening	Cervical cancer screening	HBV vaccination	Cervical cancer screening
5	FOBT for colorectal cancer	Mammography for breast cancer screening	FOBT for colorectal cancer	Raising awareness of knowledge related to primary cancer prevention
6	HBV/HCV screening during pregnancy	HBV/HCV screening during pregnancy	Cancer education for elementary, junior high, and high school students (including smoking prevention education)	Cancer education for elementary, junior high, and high school students (including smoking prevention education)
7	HBV vaccination	Cancer education for elementary, junior high, and high school students (including smoking prevention education)	Mammography for breast cancer screening	H. pylori infection screening (blood, urine, or stool test)
8	HBV/HCV screening (blood test)	H. pylori infection screening (blood, urine, or stool test)	HPV vaccination for males	Lung cancer screening (X-ray)
9	Smoking cessation treatment in clinics and support for quitting smoking in health checkups and medical settings	HPV vaccination for males	Tobacco control policies	HPV vaccination for females
10	HPV vaccination for males	HBV/HCV screening (blood test)	Raising awareness of knowledge related to primary cancer prevention	Tobacco control policies
11	Cancer risk assessment, genetic counseling, and genetic testing for patients and relatives with hereditary breast and ovarian cancer	Increasing the price of alcoholic beverages to reduce alcohol consumption	H. pylori infection screening (blood, urine, or stool test)	HBV/HCV screening (blood test)
12	Cancer education for elementary, junior high, and high school students (including smoking prevention education)	HBV vaccination	HBV/HCV screening (blood test)	HBV vaccination
13	H. pylori infection screening (blood, urine, or stool test)	Smoking cessation treatment in clinics and support for quitting smoking in health checkups and medical settings	Gastric cancer screening (X-ray or endoscopic examination)	Smoking cessation treatment in clinics and support for quitting smoking in health checkups and medical settings
14	Low-dose CT lung cancer screening for heavy smokers meeting specific criteria	Gastric cancer screening (X-ray or endoscopic examination)	Increasing the price of alcoholic beverages to reduce alcohol consumption	Gastric cancer screening (X-ray or endoscopic examination)
15	Raising awareness of knowledge related to primary cancer prevention	Raising awareness of knowledge related to primary cancer prevention		Community-wide campaigns to increase physical activity
16	Gastric cancer screening (X-ray or endoscopic examination)	Cancer risk assessment, genetic counseling, and genetic testing for patients and relatives with hereditary breast and ovarian cancer	Lung cancer screening (X-ray)	Cancer risk assessment, genetic counseling, and genetic testing for patients and relatives with hereditary breast and ovarian cancer
17	Screening for unhealthy alcohol use in adults aged 20 and over, and brief behavioral counseling to reduce it	Low-dose CT lung cancer screening for heavy smokers meeting specific criteria	Cancer risk assessment, genetic counseling, and genetic testing for patients and relatives with hereditary breast and ovarian cancer	HPV vaccination for males
18	Increasing the price of alcoholic beverages to reduce alcohol consumption	Lung cancer screening (X-ray)	Low-dose CT lung cancer screening for heavy smokers meeting specific criteria	Low-dose CT lung cancer screening for heavy smokers meeting specific criteria
19	Weight loss interventions for obese adults	Community-wide campaigns to increase physical activity	Screening for unhealthy alcohol use in adults aged 20 and over, and brief behavioral counseling to reduce it	Increasing the price of alcoholic beverages to reduce alcohol consumption
20	Environmental improvements to promote physical activity	Screening for unhealthy alcohol use in adults aged 20 and over, and brief behavioral counseling to reduce it	Smoking cessation treatment in clinics and support for quitting smoking in health checkups and medical settings	Screening for unhealthy alcohol use in adults aged 20 and over, and brief behavioral counseling to reduce it
21	Prescription of preventive medication for individuals at high risk of breast cancer, such as those with a family history	Prescription of preventive medication for individuals at high risk of breast cancer, such as those with a family history	Community-wide campaigns to increase physical activity	Environmental improvements to promote physical activity
22	Lung cancer screening (X-ray)	Weight loss interventions for obese adults	Weight loss interventions for obese adults	Weight loss interventions for obese adults
23	Community-wide campaigns to increase physical activity	Environmental improvements to promote physical activity	Prescription of preventive medication for individuals at high risk of breast cancer, such as those with a family history	Prescription of preventive medication for individuals at high risk of breast cancer, such as those with a family history

*Abbreviations*: *HBV* Hepatitis B virus, *HCV* Hepatitis C virus, *HPV* Human papillomavirus, *FOBT* Fecal occult blood test, *HBOC* Hereditary breast and ovarian cancer, *CT* Computed Tomography

## Discussion

In this study, we used the CHNRI method to select which of a number of effective interventions should be prioritized for implementation for cancer prevention in Japan. We achieved this through collaboration between researchers in the field of cancer prevention and a Patient-Public Panel organized by National Cancer Center Institute for Cancer Control, National Cancer Center Japan. To our knowledge, this is the first study to employ the CHNRI method for structured prioritization of implementation research areas in cancer prevention. Further, it is unique in that it collaboratively engaged members of the public in setting priorities for effective cancer prevention interventions through to their implementation.

Regardless of whether weighting was applied, the top five prioritized interventions were HPV vaccination for females, cervical cancer screening, FOBT for colorectal cancer screening, mammography for breast cancer screening, and tobacco control through policy measures.

The highest priority was assigned to HPV vaccination for females. This is likely influenced by the particular context surrounding HPV vaccination in Japan. Routine HPV vaccination for adolescent girls aged 12 to 16 was introduced in April 2013, and the uptake initially reached 70%. However, after the emergence of reports of persistent pain potentially linked to the vaccine, active recommendation of HPV vaccination was suspended in June 2013. The spread of misinformation regarding the HPV vaccine significantly undermined public confidence in its safety [17] and contributed to a substantial decline in vaccination uptake [18], which by fiscal year 2020 had decreased among eligible girls aged 12 to 16 to 15.9% [19]. In April 2022, the MHLW in Japan resumed active recommendation of the HPV vaccine for the first time in nine years. This resumed recommendation targeted not only girls aged 12 to 16 but also a catch-up cohort of females born between fiscal years 1997 and 2006 who had missed vaccination opportunities during the suspension period. According to a study by Yagi et al. [20], if the vaccination uptake observed in fiscal year 2022 continues in subsequent years, cumulative HPV vaccination coverage by the end of the routine schedule (i.e., end of the first year of high school) is projected to be 43.2% among girls born in FY2010 and later. This figure falls significantly short of the WHO’s target of 90% coverage required to eliminate cervical cancer (defined as an incidence rate of less than 4 per 100,000 women). Thus, uptake of the HPV vaccine in Japan remains remarkably low, making the promotion of vaccination coverage an urgent and pressing public health issue.

Nevertheless, HPV vaccination was assigned a lower priority score when evaluated in terms of feasibility. A national survey targeting women aged 16–27 and mothers of adolescents in 6th grade through 1st grade at high school year found that 26.4% expressed concern about adverse reactions to the HPV vaccine [21]. Another survey of adults across Japan revealed lower levels of awareness about HPV and knowledge of HPV vaccine efficacy, particularly among men and those with lower educational attainment [22]. These factors likely contributed to the lower priority score of HPV vaccination from the perspective of “feasibility.”

Cervical cancer screening received the second-highest priority score following HPV vaccination to females, potentially because the screening uptake in Japan remains low at 43.6%, or approximately half that of many other countries [23]. Considered together with the very low HPV vaccination coverage, improving cervical cancer screening uptake is particularly important for effective prevention of cervical cancer in the Japanese context. Of note, screening uptake among women in their early 20s is only 17.4% [24]; given the higher incidence of cervical cancer in younger women, improving screening uptake may have been seen as an urgent issue.

Colorectal and breast cancer screening also received high priority scores. This may reflect the discrepancy between the existence of organized screening programs with public financial support aimed at reducing cancer mortality and the relatively low screening uptake in Japan compared to other countries.

“Tobacco control through policy measures” was likewise regarded as a high-priority intervention, ranking fifth overall. This result may reflect Japan’s lag in implementing regulatory frameworks to improve the tobacco control environment, as noted by the WHO [25]. These include measures such as pictorial health warnings on cigarette packaging and restrictions on tobacco advertising. Tobacco control through policy measures received the highest rating for cost-effectiveness, suggesting that respondents regarded it to be a cost-effective approach to prevention.

In the present study, the top five priority scores remained unchanged regardless of weighting. This finding has also been observed in previous research [10]. This may be because, apart from the criterion of “reducing disparities,” all other evaluation criteria were considered highly important by both researchers and the Patient–Public panel, and thus the application of weighting did not alter the final results.

This study has several limitations. First, the representativeness of the Patient-Public Panel should be noted. Many of the panel members were cancer survivors or had family members with cancer, and therefore likely had greater knowledge of and interest in interventions for cancer prevention compared to the general population. In addition, their strong motivation to engage in socially contributive activities may have further enhanced their awareness and interest in cancer prevention. As a result, the findings of this study may not fully reflect the perspectives of the general public. In the future, it will be necessary to develop systems that enable the general public to participate in prioritizing the implementation of effective interventions for cancer prevention to implement. Second, there are limitations related to the evaluation criteria. While the Patient-Public Panel ranked the importance of nine evaluation criteria, the researchers assessed cancer interventions using only four of these criteria in the second survey. To more accurately reflect public perspectives, it would have been preferable to evaluate the interventions using all nine criteria, and applying weights based on their ranked importance. However, we reduced the number of criteria to four to minimize respondent burden and maintain response rates. Notably, the four selected criteria included those ranked highly by the Patient-Public Panel, namely reduction of disease burden, reduction of health disparities, and feasibility (the latter incorporating simplicity). We therefore consider that the selected criteria reflect the public’s priorities to a considerable extent. Third, we did not conduct a systematic review to compile the list of effective interventions for cancer prevention. In this study, interventions were selected based on established guidelines, including those from USPSTF, CPSTF, and the Cancer Prevention Guidelines for the Japanese Population. However, these guidelines may lag in incorporating the most recent evidence, and thus some emerging or updated findings may not have been captured. Fourth, our findings may have been influenced by potential selection bias related to the areas of expertise of the participating researchers. This study included researchers who had conducted or published cancer prevention research involving Japanese populations between January 2020 and November 2024, using either public research funding or through academic publications. Notably, this time frame overlaps with the period during which routine HPV vaccination was resumed in Japan. Consequently, there was an increase in research activity related to HPV vaccination and cervical cancer compared to other cancer types. As a result, a relatively large number of researchers with expertise in HPV vaccination and cervical cancer may have been included in the study, which could have affected the prioritization scores. Given this context, the establishment of priorities for the implementation of effective interventions for cancer prevention should not be considered a one-time effort, but should rather be conducted on a regular basis to reflect evolving evidence, policy changes, and shifts in research focus across the cancer prevention landscape. Fifth, while prioritization informed by researchers and citizens represents only one component of decision making, the final decision-making process is subject to various constraints, including resource availability, workforce capacity, and cost-effectiveness considerations. In addition, prioritization may also be influenced by contextual and social factors that were not directly assessed in this study. For example, viral hepatitis-related interventions were not highly prioritized in this study, despite their recognized public health importance. One possible explanation is that Japan has already achieved high HBV vaccination, diagnosis, and treatment [26], which may have influenced participants’ perceptions of priority. At the same time, stigma associated with viral hepatitis may also act as a barrier to testing, linkage to care, and treatment, potentially affecting perceptions of priority [27].

A key strength of this study lies in its collaborative approach with members of the public, who are the primary beneficiaries of cancer interventions, to prioritize the implementation of effective interventions for cancer prevention. In the survey involving the Patient and Public Panel, participants—unlike researchers—assigned greater importance to the criterion of reducing health disparities. This observation indicates the differing priorities and perspectives of researchers and members of the public when evaluating effective interventions for cancer prevention to implement. Accordingly, the inclusion of public perspectives in this study allowed for a more comprehensive and balanced prioritization process by capturing viewpoints that might otherwise be overlooked by researchers. Previous studies have also suggested that incorporating the views of patients and citizens is essential when setting research priorities, particularly to clarify differences in perspectives between researchers and the general public [9]. By integrating public perspectives in the prioritization process, this study has improved the potential for successful implementation of effective interventions for cancer prevention.

## Conclusions

Through a collaborative process involving researchers and citizens, this study systematically identified priority areas for implementation research in cancer prevention. Strengthening support among citizens will be important for their successful implementation. As a next step, these stakeholder-informed priorities should be communicated to policymakers to support the strategic allocation of implementation efforts. The findings of this study may help future research agendas and advancing implementation science in cancer prevention.

## Supplementary Information

Supplementary Material 1: Additional file 1. Candidate effective interventions for cancer prevention recommended for implementation in Japan: Results from the First Survey of Researchers. Detailed results of the first researcher survey.

## Data Availability

The datasets generated and/or analyzed during the current study are available from the corresponding author on reasonable request.

## Abbreviations

CHNRI: Child Health and Nutrition Research Initiative
USPSTF: United States Preventive Services Task Force
CPSTF: Community Preventive Services Task Force
HBV: Hepatitis B virus
HCV: Hepatitis C virus
HPV: Human papillomavirus
CT: Computed Tomography
FOBT: Fecal occult blood testing
MHLW: Ministry of Health, Labour and Welfare
