# Supplementary material for: Priority setting for the implementation of effective interventions for cancer prevention through collaboration among patients, the public and researchers in Japan
Source: BMC Cancer. 2026 Jun 30;26:1108. doi: 10.1186/s12885-026-16415-2 (PMC13584265; doi:10.1186/s12885-026-16415-2)
Supplement: Supplementary file 1 [file 12885_2026_16415_MOESM1_ESM.docx]

# **Additional file 1**. Candidate effective interventions for cancer prevention recommended for implementation in Japan: Results from the First Survey of Researchers

| **Category** | **Candidate Strategies** |
| --- | --- |
| **Screening and Risk Assessment** | Colonoscopy for colorectal cancer  ABC risk stratification for gastric cancer  Low-dose CT for lung cancer in non-smokers  Ultrasound screening for breast cancer  MRI-based screening and self-sampling HPV testing for cervical cancer  Targeted screening and abdominal ultrasonography for liver cancer  Targeted screening and abdominal ultrasonography for pancreatic cancer  Focused prostate cancer screening for men with family history  Risk assessment, genetic counseling, and testing for Lynch syndrome and familial adenomatous polyposis  Brain tumor screening  HTLV-1 antibody testing during pregnancy  Risk assessment based on ALDH2 polymorphisms |
| **Preventive Medical Interventions** | HPV vaccination for males  Risk-reducing surgery for hereditary breast and ovarian cancer (HBOC)  Prophylactic hysterectomy and bilateral salpingo-oophorectomy for Lynch syndrome  Chemoprevention for hereditary colorectal cancer  Aspirin use for colorectal cancer prevention |
| **Lifestyle Modification** | Smoking cessation support (e.g., legal restrictions, cessation counseling in healthcare settings)  Countermeasures against unhealthy drinking (e.g., education on acute alcohol intoxication and alcohol use disorder)  Dietary improvement (e.g., balanced diet, increased vegetable intake, specific nutrient supplementation) and supportive environments  Guidance regarding consumption of extremely hot or spicy foods |
| **Education and Information Dissemination** | Comprehensive health education (including sexual and infection prevention education)  School-based education on HPV vaccination and cervical cancer screening  Environmental support for implementing cancer prevention policies  Prevention of medical misinformation (e.g., dissemination of evidence-based information, legal measures against harmful content)  Cancer education programs |
